# Changes in food behaviours during the first COVID‐19 pandemic lockdown in Australia

**DOI:** 10.1111/1747-0080.12921

**Published:** 2024-12-16

**Authors:** Aliyah Palu, Juliette Crowther, Ashleigh Chanel Hart, Joseph Alvin Santos, Emalie Rosewarne, Simone Pettigrew, Annet C. Hoek, Kathy Trieu, Jacqui Webster

**Affiliations:** ^1^ The George Institute for Global Health UNSW Sydney Australia; ^2^ Department of Business Economics, Health, and Social Care (DEASS) University of Applied Sciences and Arts of Southern Switzerland (SUPSI) Switzerland

**Keywords:** COVID‐19, eating behaviour, food planning, food preparation, food selection, nutrition

## Abstract

**Aim:**

The objective of this study was to explore changes in Australian consumer food behaviours during COVID‐19 public health restrictions (lockdown), to provide insights into how this unforeseen crisis event affected dietary behaviour patterns.

**Methods:**

An online cross‐sectional survey was conducted in September 2020 with a nationally representative sample of the Australian adult population. Participants were asked to complete questions about (1) meal planning and food purchasing and (2) barriers to cooking, before and during a national‐wide COVID‐19 lockdown in early 2020. A survey‐adjusted logistic regression analysis was used to identify food behaviour changes resulting from the lockdown.

**Results:**

A total of 4022 respondents completed the survey. Overall, food behaviour changes were found to be small and mostly positive. Eight of the 14 meal planning and purchasing related behaviours improved. This included more frequent meal planning and more time to be able to cook. However, not all changes were healthier, with more respondents reporting that they cooked meals at home using healthy ingredients less frequently during the lockdown.

**Conclusion:**

These findings demonstrate that people are willing and able to make some positive changes when they have time but that more work needs to be done to ensure that the improvements in food literacy result in healthier meals. Further consideration also needs to be given to how we can embed and amplify these positive changes into everyday habits now that public health restrictions have lifted and Australia is in post‐lockdown reality.

## 1 INTRODUCTION

The COVID‐19 pandemic, and the subsequent public health restrictions (hereafter called lockdowns) that occurred around the world, had widescale impacts on food environments and individuals' lifestyles.^1^ These changes significantly affected individuals' food behaviours, including how they shopped, chose food products, prepared meals, ate, and handled food waste.^2^ Food behaviour is a highly important determinant of nutritional intake.^3^ Consuming a balanced diet can contribute to sustaining both physical and mental well‐being.^3^ Conversely, unhealthy eating habits, including skipping meals and consuming nutrient deficient meals, are a major risk factor for diet‐related noncommunicable diseases (NCDs)^4^ including heart disease, the leading cause of death in Australia and globally.^5^, ^6^ Food behaviour is described as food choice and motives, and is often the term used when understanding the relationship between diet and diet‐related NCDs.^7^ Within the broader concept of food behaviours is the idea of food literacy, which encompasses nutrition skills and knowledge such as cooking, food prep and food skills.^8^ Both food behaviours and food literacy is highly contextual and shaped by personal, social and environmental factors.^3^

In March 2020, Australia entered its nationwide lockdown to control the spread of COVID‐19.^9^ The lockdown lasted about 6 weeks^10^ and mandated people to stay at home, and the closure of non‐essential businesses and facilities, including pubs, clubs, gyms, cinemas, entertainment venues, and religious gatherings.^9^ Consumers had reduced opportunity to eat outside the home and spent more time at home.^2^ The food environment was dramatically changed as food service outlets such as restaurants and cafes were restricted to takeaway and/or home delivery. Although retail outlets such as supermarkets and convenience stores remained open due to their essential nature,^9^ there were major demand and supply issues as consumers began panic‐buying non‐perishable food items, and delivery schedules were abandoned due to drivers contracting COVID‐19. Consumers faced empty shelves, quantity limits per transaction on particular food items, and product unavailability.^11^ This led to dedicated supermarket shopping hours for pensioners and people with disabilities to ensure they had opportunity to conduct their grocery shopping.^12^ These drastic changes during the COVID‐19 lockdown provide a unique opportunity to explore how food behaviours were affected.

International research into the impacts of COVID‐19 lockdowns on people's food behaviours have produced mixed findings. Most studies found food consumption behaviours deteriorated, including increases in snacking, and total daily food intake.^13^, ^14^, ^15^, ^16^, ^17^ However, several studies also found positive changes in healthy food behaviours including increases in healthy meal planning, food purchasing, and food preparation.^1^, ^18^, ^19^, ^20^ These findings are also reflected in Australian studies, where improved cooking skills and confidence, meal planning and purchasing skills, as well as increased home cooking and experimentation in the kitchen, and consumption of family meals were reported during COVID‐19 lockdowns.^21^, ^22^ These healthy food behaviours are essential to food literacy, which is the collection of knowledge, skills, and behaviours required to plan, manage, select, prepare, and eat a healthy diet.^23^ Higher food literacy scores are associated with healthier diet quality,^24^ and an ability to revise and adapt dietary behaviours when disruptive life events and circumstances occur.^23^

Research into how food behaviours were affected during COVID‐19 lockdowns is helpful for understanding how to improve food literacy skills and national dietary intakes. The aim of this survey was to assess how food behaviours relating to food literacy were affected by the first COVID‐19 lockdowns in Australia.

## 2 METHODS

An online cross‐sectional survey of Australian adults was conducted during September 2020. The study was performed in compliance with the Helsinki Declaration guidelines. This work was done in accordance with the STROBE guidelines for observational studies. Ethics approval was obtained through the Human Research Ethics Committee of the University of New South Wales (HC200447). Participation in the research was voluntary. At the beginning of the study, each respondent was informed of the study objective and context and provided their written informed consent regarding privacy and information management policies.

Accordingly, a probability proportional to size sampling method was used to recruit a nationally representative sample of 4022 adults aged 18 years or older living in Australia, stratified by age, sex, and state using the most recent census data.^25^ Respondents were recruited from an International Organisation for Standardisation‐ certified commercial online research panel in Australia (Kantar Profiles Healthcare).^26^ Kantar Profiles Healthcare network provides pre‐profiled consumers a platform to contribute to research and be paid in Kantar credit points.^27^ The network monitors recruitment to ensure fraud, location, and duplication is detected.^27^

**FIGURE 1:**
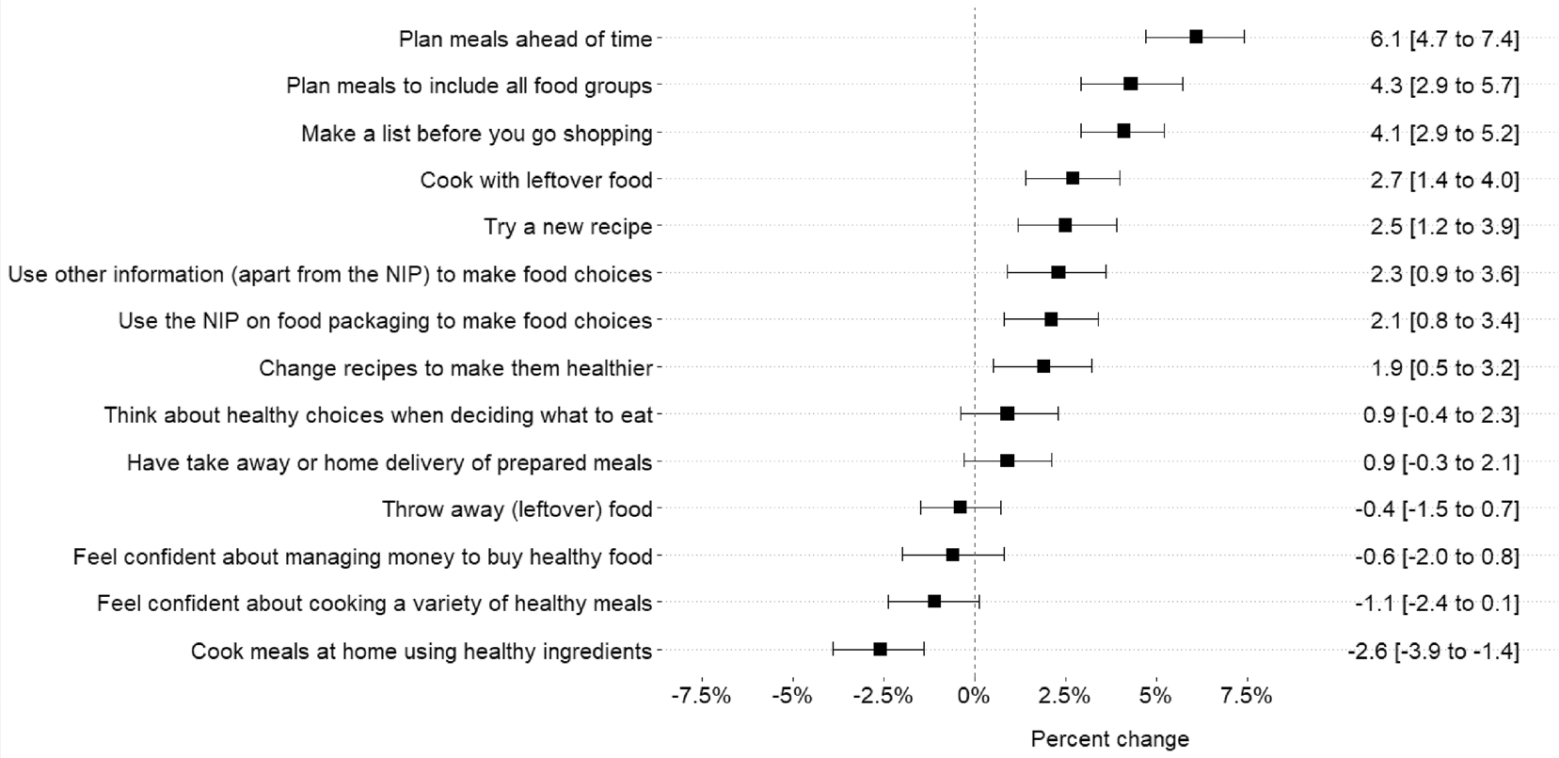
Percentage changes in frequency of engaging in food behaviours (*n* = 4022). A positive change indicates participants engaged in the behaviour more frequently during compared to before the lockdown, whilst a negative change indicates less frequent engagement.

The survey instrument was based on a validated tool used in a multinational survey that investigated the impact of social distancing policies during the COVID‐19 pandemic on food behaviours and food literacy.^1^ The survey components described in this paper included two sections relating to (1) Food literacy: Meal planning and purchasing and (2) Food behaviours: Barriers to cooking which were adapted from the multinational survey.^1^ The meal planning and purchasing section contained 14 items relating to planning (e.g. plan meals to include all food groups), selecting (e.g. use the nutrient information panel (NIP) on food packaging to make food choices), and preparing foods (e.g. try a new recipe) (see Figure 1). The barriers to cooking section contained five items on cooking barriers, including time (I didn't have time to cook) and money (I didn't have funds for the foods or ingredients I needed) (see Table 3). Respondents were asked to report how often they performed each food behaviour both before and during lockdown. The survey questions made it clear that respondents specifically focus on the national lockdown that occurred at the start of the COVID‐19 pandemic. All items were rated on a seven‐point frequency scale with answer categories.

The responses to the questions were dichotomised as: never, very rarely, rarely, sometimes versus frequently, very frequently, every time. A survey‐adjusted logistic regression model accounting for the matched nature of the response (i.e. repeated questions for *before* and *during* the lockdown) was used to determine changes from before to during the lockdown. The adjusted proportions before and after the lockdown, and the percent change over time, were calculated from the fitted model using the margins command. Subgroup analyses were conducted by sex (males, females) and age group (18–44 years, 45–64 years, and 65+ years). Analyses were conducted using Stata software version 15.1 for Windows (StataCorp LLC, TX, USA). Figures were generated using R version 1.2.1572.

## 3 RESULTS

A total of 4022 participants completed the survey, 51% of whom identified as female and 49% identified as male, with a mean age of 48 years (Table 1). The majority of the sample was fully or partially responsible for grocery shopping (95.9%) and for cooking or preparing food (87.5%) in their household.

**TABLE 1:** Socio‐demographic characteristics of survey participants (*n* = 4022).

Study participant characteristics	*n* (%)
Sex	
Male	1970 (49.0)
Female	2052 (51.0)
Age (years)	
18–44	2044 (50.8)
45–64	1224 (30.4)
65+	754 (18.7)
Education	
Non‐tertiary	2149 (53.6)
Tertiary	1858 (46.4)
Household grocery shopping responsibility	
Main person	3149 (78.3)
Not the main person	165 (4.1)
Shared responsibility	708 (17.6)
Household cooking or food preparation responsibility	
Main person	2783 (69.2)
Not the main person	503 (12.5)
Shared responsibility	736 (18.3)

Eight of the 14‐meal planning and food purchasing related behaviours, were found to have increased and one decreased in frequency during the lockdown. There was a significant increase in planning behaviours post‐lockdown compared to pre‐lockdown. For example, respondents on average reported doing more planning of meals ahead of time, planning meals to include all food groups, making a list before going shopping, cooking with leftover foods, trying a new recipe, using nutrient information panel (NIP) or other information to make food choices, and changing recipes to make them healthier during the lockdown (Figure 1). However, not all changes were healthier, with people less frequently reporting cooking meals at home using healthy ingredients, during the lockdown. No significant changes were found for other meal planning and food behaviours. These were: Think about healthier choices when deciding what to eat, have take‐away or home delivery of prepared meals, throw away leftover food, feel confident about managing money to buy healthy food, feel confident about cooking a variety of healthy meals.

Changes in subgroups by age and sex reflected these trends. However, in the 65+ age group five of the eight behaviours that increased in frequency did not reach significance (Table 2). These were: planning meals to include all food groups, trying a new recipe, using other information (apart from the NIP) to make food choices, using the NIP on food packaging to make food choices, and changing recipes to make them healthier.

**TABLE 2:** Percentage change (95% CI) in meal planning and food purchasing behaviour frequency reported BEFORE and DURING lockdown measures.

Meal planning and food purchasing behaviour	Total sample (*n* = 4022)	Sex		Age group		
		M	F	18–44 years	45–64 years	65+
Performed *frequently or very frequently or every time*	% (95% CI)	% (95% CI)	% (95% CI)	% (95% CI)	% (95% CI)	% (95% CI)
Plan meals ahead of time	6.1 (4.7, 7.4)*	5.1 (3.3, 6.9)*	7.0 (5.1, 8.9)*	5.8 (3.8, 7.8)*	6.8 (4.5, 9.1)*	5.5 (3.0, 8.1)*
Make a list before you go shopping	4.1 (2.8, 5.3)*	3.0 (1.2, 4.7)*	5.1 (3.5, 6.7)*	5.3 (3.4, 7.3)*	3.0 (1.3, 4.8)*	2.2 (0.7, 3.8)*
Plan meals to include all food groups	4.3 (2.9, 5.7)*	3.5 (1.5, 5.5)*	5.0 (3.1, 6.9)*	4.9 (2.8, 7.1)*	4.3 (1.9, 6.6)*	2.4 (−0.2, 4.9)*
Think about healthy choices when deciding what to eat	0.9 (−0.4, 2.3)	1.1 (−0.8, 3.0)	0.7 (−1.1, 2.6)	1.4 (−0.6, 3.5)	0.3 (−1.8, 2.5)	0.5 (−1.9, 3.0)
Feel confident about managing money to buy healthy food	−0.5 (−1.9, 0.7)	0.3 (−1.6, 2.3)	−1.5 (−3.3, 0.4)	−0.5 (−2.8, 1.6)	−1.1 (−3.3, 1.0)	0.3 (−1.9, 2.5)
Use the NIP on food packaging to make food choices	2.1 (0.8, 3.4)*	2.2 (0.5, 4.0)*	1.9 (0.2, 3.7)*	2.2 (0.2, 4.2)*	3.3 (1.3, 5.2)*	0.0 (−2.5, 2.5)
Use other information apart from NIP to make food choices	2.2 (0.9, 3.6)*	2.2 (0.3, 4.1)*	2.3 (0.3, 4.1)*	3.0 (0.9, 5.1)*	1.1 (−1.0, 3.2)	2.0 (−0.5, 4.6)
Cook meals at home using healthy ingredients	−2.6 (−3.9, −1.4)*	−2.6 (−4.5, −0.7)*	−2.6 (−4.4, −0.9)*	−1.2 (−3.3, 0.8)	−3.2 (−5.2, −1.2)*	−5.4 (−7.4, −3.3)*
Feel confident about cooking a variety of healthy meals	−1.1 (−2.4, 0.1)	−1.0 (−2.9, 0.8)	−1.2 (−2.9, 0.5)	0.1 (−1.9, 2.1)	−2.0 (−4.1, −0.07)*	−2.9 (−4.9, −0.9)*
Try a new recipe	2.5 (1.1, 3.8)*	2.3 (0.4, 4.2)*	2.6 (0.7, 4.6)*	4.8 (2.7, 7.0)*	1.1 (−1.1, 3.3)	−1.7 (−4.0, 0.6)
Change recipes to make them healthier	1.8 (0.5, 3.2)*	1.9 (−0.0, 3.8)	1.8 (−0.1, 3.7)	0.9 (−1.2, 3.0)	3.2 (1.1, 5.4)*	2.2 (−0.4, 5.0)
Have take‐away or home delivery of prepared meals	0.8 (−3.2, 2.1)	1.1 (−0.6, 2.8)	0.7 (−0.9, 2.3)	0.3 (−1.7, 2.4)	1.3 (−0.4, 2.9)	1.7 (0.2, 3.1)*
Cook with leftover food	2.7 (1.4, 4.0)*	2.2 (0.3, 4.0)*	3.2 (1.3, 5.1)*	2.8 (0.7, 4.8)*	2.4 (0.2, 4.6)*	3.0 (0.3, 5.7)*
Throw away (leftover) food	−0.4 (−1.4, 0.7)	0.1 (−1.5, 1.7)	−0.8 (−2.3, 0.6)	−1.0 (−2.8, 0.8)	0.3 (−1.3, 1.8)	0.1 (−1.2, 1.5)

*Note*: A negative value indicates a decrease in frequency of the behaviour whilst a positive value indicates an increase in frequency of the behaviour.

* Logistic regression *p* < 0.05.

**FIGURE 2:**
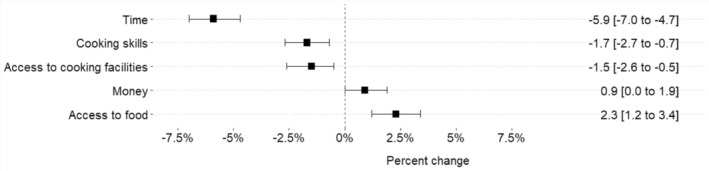
Percentage changes in frequency (% [95% CI]) of experiencing barriers to cooking during compared to before the lockdown (*n* = 4022). A negative percentage indicates that this was experienced less often as a barrier during compared to before the lockdown.

Time, cooking skills, and access to cooking facilities were experienced less frequently as barriers during the lockdown compared to pre‐lockdown (Figure 2). Access to food was experienced as a barrier more frequently (all *p* < 0.05). Changes in subgroups by age and sex generally followed the same trends (Table 3).

**TABLE 3:** Percentage change (95% CI) in experiencing barriers to cooking BEFORE and DURING lockdown measures reported by respondents.

Barriers to cooking	Total sample (*n* = 4022)	Sex		Age group		
		M	F	18–44	45–64	65+
Experienced *frequently or very frequently or every time*	% change over time (95% CI)	% change over time (95% CI)	% change over time (95% CI)	% change over time (95% CI)	% change over time (95% CI)	% change over time (95% CI)
Time (I didn't have time to cook)	−5.8 (−7.0, −4.7)*	−3.8 (−5.5, −2.2)*	−7.8 (−9.5, −6.2)*	−9.3 (−11.3, −7.4)*	−2.9 (−4.7, −1.1)*	−1.2 (−2.2, −0.2)*
Cooking skills (I couldn't cook)	−1.7 (−2.7, −0.7)*	−1.9 (−3.4, −0.4)*	−1.5 (−2.8, −0.2)*	−1.3 (−3.0, 0.4)	−1.6 (−3.1, −0.1)*	−2.9 (−4.5, −1.4)*
Money (I didn't have funds for the foods or ingredients I needed)	0.9 (−0.03, −1.9)	1.1 (−0.4, 2.6)	0.7 (−0.5, 1.9)	1.4 (−0.2, 3.0)	1.2 (−0.3, 2.7)	−0.8 (−1.9, 0.4)
Access to food (I didn't have access to food or ingredients I needed)	2.3 (1.2, 3.4)*	1.6 (0.05, 3.2)*	2.9 (1.5, 4.4)*	3.5 (1.7, 5.3)*	2.1 (0.6, 3.7)*	−0.8 (−2.4, 0.7)
Access to cooking facilities (I didn't have access to the cooking facilities I needed)	−1.5 (−2.5, −0.5)*	−1.1 (−2.6, 0.5)	−1.9 (−3.3, 0.6)*	−1.5 (−3.2, 0.1)	−1.2 (−2.8, 0.4)	−1.8 (−3.6, −0.1)*

*Note*: A negative value indicates a decrease in experiencing the barrier whilst a positive value indicates an increase in experiencing the barrier.

* Logistic regression *p* < 0.05.

## 4 DISCUSSION

This Australian survey found several areas of change occurred in food behaviours and food literacy during the national COVID‐19 lockdown. Whilst most behaviours moved in a positive direction, including respondents more frequently planning meals ahead of time, changing recipes to make them healthier, and checking labels before selecting foods, some behaviours related to healthy food such as cooking meals at home using healthy ingredients, moved in a negative direction.

Our findings show that there were small positive changes in food behaviours, which broadly reflect those of Grunert et al.^18^ study in 10 European countries. This study found that 60% of consumers maintained similar food behaviours during the pandemic, indicating the behaviours were habitual and enduring, despite varying levels of stringency in social restrictions. Across the remaining 40% of consumers, most reported making positive changes in their food behaviours, including more enjoyment in cooking and eating and more mindful eating. De Backer similarly reported increases in planning, selecting, and preparing healthy foods for men and women in response to the COVID‐19 pandemic in an observational study across 38 countries.^1^ Our findings also align with other Australian literature highlighting that Australians developed positive food behaviours, tried new recipes, and were cooking from scratch.^21^, ^22^ These behaviours relating to meal planning, selecting, and preparing are important indicators of food literacy and are linked to improved health outcomes.^28^, ^29^

Reported increased availability of time may have contributed to increased meal planning behaviours as respondents reported that time was less of a barrier to cooking at home. Meal planning is reportedly one of the most effective strategies for positively impacting healthy living and diets.^28^, ^29^ These changes may also be attributed to the lockdown restricting consumers' outings for essential purposes only, including not being permitted to dine in cafes and restaurants, and thus grocery shopping became a more deliberate activity. This is in line with a state‐level survey of Tasmanian adults that found 26% of those surveyed were shopping more deliberately. One respondent was quoted as saying: “we are now more likely to be making shopping lists and menu planning so we can shop less often and faster”.^30^ The finding that respondents reported cooking meals at home using healthy ingredients less often during the lockdown which contrasts with otherwise mostly positive changes that could be attributed to consumers cooking at home using *unhealthy* ingredients. Increased baking (which may include unhealthier foods such as cakes, pastries, pizzas, or pies) at home during lockdowns has been observed in other studies.^13^, ^18^, ^21^, ^31^, ^32^ A survey by the Australian Institute of Family Studies found almost half of respondents reported doing more baking or other skill‐based activities during the lockdown.^31^ Other studies also found that people were cooking at home more often,^13^, ^21^, ^32^, ^33^, ^34^ had more time to cook at home,^2^, ^13^, ^21^, ^33^, ^34^ were seeking new recipes,^2^, ^13^, ^21^, ^34^ and some found that most of these changes were associated with desires to use and consume healthier ingredients, such as fresh fruits and vegetables,^13^, ^32^ and to minimise food waste.^2^, ^13^, ^18^, ^32^, ^34^ Whilst our study reflects some of these positive changes, our study did not measure whether people were more likely to cook at home during lockdown or consumer motivations for cooking at home. Future research could extend work in this area by exploring whether decreasing time barriers improve the healthiness of meal planning, selecting, and cooking at home.

Improving consumer food literacy has important implications for public health in Australia. Food literacy is relevant to reducing diet‐related NCDs, including obesity and reducing the risk of COVID‐19 severity and death. This study found Australian consumers may adopt healthier dietary behaviours and improve food literacy when experiencing a disruptive event such as the lockdown. Given the pandemic has allowed people to transition to more flexible work arrangements post‐lockdown, it is important to consider the broader context of this new reality. Recent international studies highlight other potential negative health impacts of working from home during COVID‐19 lockdowns, including physical and mental health issues (linked to worsening diets,^35^ less physical activity, increase in daily sitting and screen times and sleep disturbances).^35^, ^36^ Therefore, strategies that focus on improving food literacy irrespective of time availability are needed.

A key strength of this study is that rather than using convenience sampling like other web‐based surveys,^13^ it used a probability proportional to size sampling method that provided a large and nationally representative sample.^25^ However, only those with internet access could participate. Another strength of this study was the use of an existing survey tool that was adapted from a larger multinational survey.^1^ However, a limitation was that the instrument relied on self‐reported data in response to survey questions simultaneously asking about two timepoints (before and during the lockdown), which may have introduced recall bias. Furthermore, data were collected 5 months after the national lockdown was implemented, when states in Australia were experiencing different lockdown responses to COVID‐19.

Whilst this study demonstrated small changes in food behaviours during the first COVID‐19 lockdowns during early 2020, not all changes were positive. More work needs to be done to ensure that the improvements in food literacy result in healthier meal meals including through increasing confidence for people to budget for and prepare healthier meals. Further consideration also needs to be given to how we can embed and amplify the positive changes into everyday habits now that the lockdowns have ended.

## FUNDING INFORMATION

This study was funded by The George Institute's Thought Leadership Program. JW is supported by a National Health and Medical Research Council Investigator Grant Level 2 and receives additional funding from the National Health and Medical Research Council and the Ian Potter Foundation. KT was supported by an Early Career Fellowship (APP1161597) from the National Health and Medical Research Council of Australia (NHMRC) and a Postdoctoral Fellowship (Award ID 102140) from the National Heart Foundation of Australia.

## CONFLICT OF INTEREST STATEMENT

The authors have no conflicts of interest to disclose.

## ETHICS STATEMENT

All procedures relevant to study respondents were approved by the University of New South Wales Human Research Ethics Committee (approval number HC200447).

## Data Availability

The data that support the findings of this study are available from the corresponding author upon reasonable request.
